# Tuning the Tumor Myeloid Microenvironment to Fight Cancer

**DOI:** 10.3389/fimmu.2019.01611

**Published:** 2019-07-25

**Authors:** Nadine S. Jahchan, Adriana M. Mujal, Joshua L. Pollack, Mikhail Binnewies, Venkataraman Sriram, Leonard Reyno, Matthew F. Krummel

**Affiliations:** ^1^Pionyr Immunotherapeutics, South San Francisco, CA, United States; ^2^Department of Pathology, University of California, San Francisco, San Francisco, CA, United States; ^3^ImmunoX Initiative, University of California, San Francisco, San Francisco, CA, United States

**Keywords:** tumor micoenvironment, macrophage, tumor associated macrophage (TAM), immune checkpoint blockade (ICB), dendritic cell (DC), myeloid cells, myeloid tuning, monocytes

## Abstract

The tumor microenvironment (TME) of diverse cancer types is often characterized by high levels of infiltrating myeloid cells including monocytes, macrophages, dendritic cells, and granulocytes. These cells perform a variety of functions in the TME, varying from immune suppressive to immune stimulatory roles. In this review, we summarize the different myeloid cell populations in the TME and the intratumoral myeloid targeting approaches that are being clinically investigated, and discuss strategies that identify new myeloid subpopulations within the TME. The TME therapies include agents that modulate the functional activities of myeloid populations, that impact recruitment and survival of myeloid subpopulations, and that functionally reprogram or activate myeloid populations. We discuss the benefits, limitations and potential side effects of these therapeutic approaches.

## Introduction

The tumor microenvironment (TME) consists of a cellular multitude including fibroblasts, endothelial cells, and immune cells from the lymphoid and myeloid lineage (1–3). The TME shapes the growth of tumor cells and influences responses to therapies (4). In cancer patients, the immune system fails to suppress tumor growth in part due to the presence of active immune checkpoints or “brakes,” that usually result in the suppression of T-cell function (5, 6). CTLA-4 was the first immune checkpoint identified on T-cells in 1996 (7) and led to the development of the anti-CTLA-4 antibody Ipilimumab that is now approved in the clinic (8). PD-1 (9) was the second immune checkpoint identified (10) and led to the development of multiple anti-PD-1 and anti-PD-L1 monoclonal antibodies that are now approved therapies (11, 12). These Immune Checkpoint Blockade (ICB) therapies mainly function by re-engaging the immune system to promote anti-tumor activity. In the clinic, ICB therapies have shown profound clinical benefits and durable responses in a subset of patients in multiple tumor indications, including metastatic melanoma, NSCLC, and renal cell carcinoma (13). Only about 25% of patients across all indications respond to ICB therapies, highlighting the importance for additional therapies to treat the remaining non-responsive patients (14). There is currently a major effort to develop therapies that block additional immune inhibitory pathways (e.g., TIM3, LAG3, IDO, VISTA, and KIR) or that activate immune co-stimulatory receptors (e.g., CD40, GITR, ICOS, CD137, and OX40) (15). To date, these second generation immune-therapies have yet to yield significant clinical efficacy beyond anti-PD-1, anti-PD-L1, or anti-CTLA-4 therapies. For instance, IDO-1 inhibitors failed to provide benefit as monotherapy in Phase I/II clinical trials (16, 17) and in combination with anti-PD-1 therapy in a Phase III clinical trial in advanced melanoma patients (18), highlighting the challenges to understand the biology of this drug target and to explore further combination therapies in the clinic.

Both the lack of progress in next-generation ICB agents targeting the T cells as well as identification of resistance and regulatory pathways beyond T cells in the TME has renewed interest in identification of novel targets in the TME. Profiling the immune cells in the TME of patients with advanced techniques demonstrated significant differences in the immune infiltrates and composition of the TME within patients from the same tumor types, especially in cells from the myeloid lineage (19–23). The intratumoral myeloid cells in the TME are heterogeneous in nature and include mononuclear cells (monocytes, macrophages, dendritic cells), and polymorphonuclear granulocytes (3, 19, 24, 25). In normal tissues, these myeloid cells assist in damage repair and provide a first line of defense against dangers such as pathogens and viruses. They are not uniform, either in form or function, presumably to ensure versatile responses to the diverse challenges faced in normal and disease physiology. In the TME, they can either suppress or promote anti-tumor immunity and play an important role in phagocytosis and antigen presentation to T-cells (24, 26, 27). For instance, myeloid inhibitory cells such as tumor-associated macrophages (TAMs) can limit responses to chemotherapy, irradiation, and angiogenic inhibitors (28–30). In contrast, stimulatory myeloid cells such as migratory dendritic cells (DCs) are critical for eliciting potent anti-tumor T-cell responses, and patients with higher migratory DCs have significantly increased overall survival (19, 20, 24, 26, 27, 31). Despite the potential to mediate antitumor effector T cell immunity, however, steady-state DC populations also maintain peripheral T cell tolerance (32, 33) and these baseline homeostatic processes may compromise their stimulatory capacity in some patients (34, 35). Therefore, strategies to target specific myeloid populations and cellular programs in the clinic have attracted considerable attention from many companies, and multiple drug agents are currently being evaluated in the clinic. In this review, we describe the myeloid subpopulations in the TME and summarize the different myeloid tuning strategies to target these cells.

## Myeloid Subpopulations in the Tumor Microenvironment

**Figure 1:**
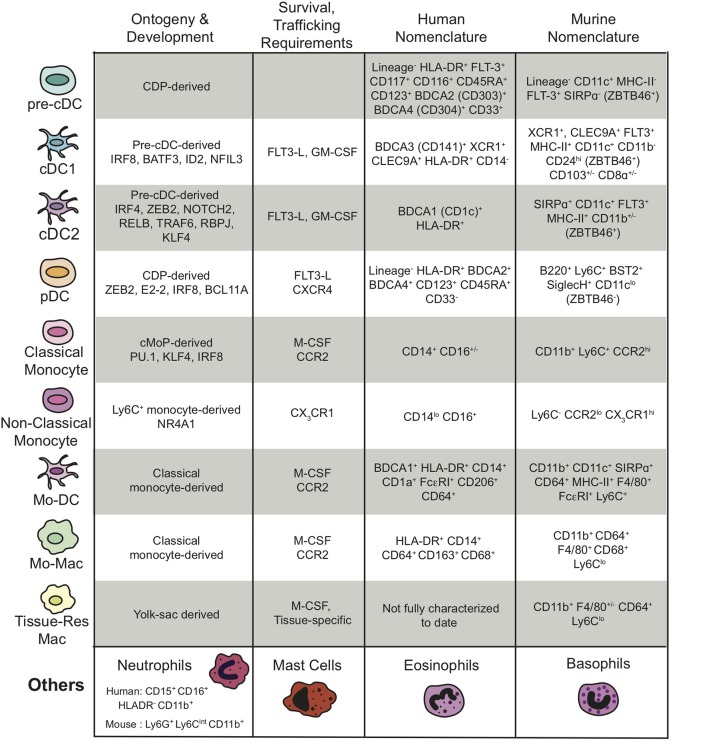
Summary of the different subtypes of myeloid cells present in the TME, their ontogeny and development, their survival and trafficking requirements, and their human and mouse nomenclatures including transcription factors and cell surface markers. The “Others” myeloid cells in the last row represent the granulocytes. “Lineage^−^” is defined as CD3^−^CD14^−^CD16^−^CD19^−^ for the human nomenclature and CD3^−^NKp46^−^B220^−^ for the murine nomenclature.

The myeloid cell populations within the TME, their ontogeny and development, the key chemokines required for their trafficking and survival, as well as the gene products that are used by many researchers to define the various myeloid populations in humans and mice are outlined in Figure 1 and discussed in more detail below.

### Macrophages

The most abundant myeloid population in tumors are generally TAMs (27, 36). Though an inclusive term, TAMs have heterogeneous ontogeny and can broadly be classified on this basis as monocyte-derived macrophages “mo-Macs” or yolk-sac derived tissue-resident macrophages (29, 30, 36, 37). While high frequencies of TAMs are generally associated with poor prognosis in a wide variety of cancer indications, correlations between high TAM density and improved survival have also emerged (30, 38, 39). These discordant observations raise questions about whether there are qualities of TAMs that can make them beneficial to an adaptive response, and also reinforce the need for markers to rigorously distinguish TAMs from distinct origins, distinct phenotypes, as well as from other myeloid populations (Figure 1). Like some of their other myeloid counterparts, TAMs take on distinct activation states. Although it trivializes the diversity *in vivo*, TAMs are often reduced to existing in binary states of classical (“M1”) or alternative (“M2”) activation based on *in vitro* studies that skewed macrophage differentiation with the single chemokines IFN-γ vs. IL-4, respectively. Although more reductionist than what occurs *in vivo*, these two cellular profiles exemplify the possibility of polarized transcriptional and secretory programs, and those in turn may explain conflicting correlations in patient outcome (40). For instance, “M2” signatures, which sometimes correlate with poor prognosis, tend to be anti-inflammatory and associated with tissue remodeling and wound-healing processes (30, 38). However, it is now clear that activation and polarization of macrophages consist of a range of non-terminal phenotypes rather than two binary states and there are multiple factors contributing to their intratumoral and intertumoral heterogeneity, such as the anatomical location, cancer subtype, and exposure to a myriad of environmental factors corrupting the TAMs to exist in a katzenjammer state (30, 38, 41, 42).

The heterogeneity of macrophages may be due to lineage-imprinted differences between mo-Macs and tissue-resident macrophages (30, 37, 43, 44). While some tissue-resident macrophages express tissue-specific markers (45), recent advances have improved separation of mo-Macs and tissue-resident macrophages in the TME (36, 37). In these studies, tissue-resident macrophages exhibited a stronger “M2” profile (36) and took on a wound repair program (37) while mo-Macs exhibited the ability to prime CD8+ T cells, although these experiments were performed *in vitro* with pulsed antigen, bypassing normal cross-presentation machinery (37). RNA-sequencing (RNA-seq) analyses of breast and endometrial cancer TAMs in comparison with FACS sorted tissue-resident macrophages from normal tissues confirmed the existence of tissue-specific niches that influence macrophage and TAM profiles irrespective of their common precursor cells (45). A better understanding of macrophage origin and heterogeneity is vital when exploring the effects of targeting the macrophage population within the TME. Recent studies using single-cell profiling by RNA-seq suggest a more complex heterogeneity and plasticity of macrophages that could further affect tumor development and responsiveness to immunotherapy (21–23).

### Dendritic Cells

Conventional DCs (cDCs) similarly exhibit diversity, broadly delimited as cDC1 and cDC2, with commitment to each occurring early in specific precursor populations, called pre-cDCs (46) and the two mature classes corresponding to differential transcription factor requirements and having functional specialization (47–49). Pre-cDCs are detectable in the blood, lymphoid, and non-lymphoid tissue, and can also be found in the TME (50). Although cellularity may vary, both cDC1s and cDC2s can be found in mouse and human tumors (21, 27, 51) and take on distinct roles in the priming of anti-tumor T cells. cDCs, particularly cDC1s, require FLT3-ligand (FLT3-L) for development and *in situ* proliferation, as well as GM-CSF for survival in peripheral tissue (52). Although there have been reports of some cancers producing GM-CSF (53), the origin of these cytokines in the TME is largely uncharacterized. Notably, recent data suggests that natural killer cells act as a rich source of FLT3-L in the TME (20).

cDC1s excel at antigen cross-presentation and are critical for initiating CD8^+^ T cell responses across a number of immunological settings, including tumor models (27, 51, 54). In mice, cDC1s have two major subclasses: lymphoid tissue resident CD8a^+^ DCs and non-lymphoid tissue (NLT) migratory CD103^+^ DCs, which are strikingly similar to one another transcriptionally and share expression of the chemokine receptor XCR1 (49, 51, 55). Together cDC1s depend on transcription factors IRF8 (49) and BATF3 (54) for development, although strict requirements between the subsets may differ (48). Although genetic models eliminating these genes are useful for broad depletion of cDC1s (54), more recent use of mixed bone marrow chimeras demonstrated a specific and critical role for CCR7^+^ CD103^+^ DCs in migration and initiation of CD8^+^ T-cells responses in tumor-draining lymph nodes (LNs) (26, 51). In addition to outperforming the other DC subset at cross-presentation, tumor cDC1s are a primary producer of IL-12 (27), which contributes to CD8^+^ T-cell proliferation and effector function and is associated with higher rates of responsiveness to chemotherapy (56). Furthermore, cDC1s exert potent anti-tumor activity in the TME despite being an extremely rare population (27). Tumor cDC1 production of CXCL9 and CXCL10 can recruit activated T- cells to the TME (57) where local cDC1 re-stimulation of T-cells support anti-tumor activity (27). Although the mechanistic requirements and consequences of DC re-activation are still not well-understood, tumor cDC1s may promote higher T-cell motility and contact with cancer cells (20, 57, 58).

In contrast to cDC1s, cDC2s typically preferentially activate CD4^+^ T-cells through presentation of peptides on MHC-II, express SIRPα, and are dependent on the transcription factor IRF4 (49, 52). Despite this overarching classification, cDC2s encapsulate a great degree of heterogeneity (55). While historically cDC2s have largely been identified as CD11b^+^ DCs (47), dermal cDC2s do include a CD11b^hi^, and CD11b^lo^ KLF4-dependent population (59), highlighting the advantage of using SIRPα as a defining marker. Another complicating feature of cDC2s is that they share many surface markers with monocytes and macrophages (e.g., CD11b, CD11c, SIRPα, CX3CR1, CCR2, CD14). While this overlap has made it difficult to precisely define and isolate cDC2s, additional markers including CD64, MERTK, and Ly6C have been proposed to selectively identify macrophages and monocytes (60). ZBTB46 has also emerged as a cDC lineage-restricted transcription factor and may help to clarify ontogeny (61). In humans, cDC2s are best aligned with the CD1c^+^ (BDCA1^+^) subset found in the blood and various tissues including tumor (35, 62, 63) and comprise at least two subset populations as revealed by recent single-cell RNA-sequencing analysis (35, 64).

### Inflammatory DCs

Although cDCs are tautologically pre-cDC-derived, monocytes can be recruited to sites of inflammation and differentiate into mo-DCs, also called inflammatory or iDCs, in response to a number of infectious or adjuvant agents (65). Monocyte ontogeny is primarily demarcated by CCR2-dependency and surface markers, and transcriptional profiling of skin cell populations revealed that mo-DCs exhibit a similar gene signature to CD11b^+^ cDCs (60). In some cases, mo-DCs may substitute for cDCs functionally or shape T-cell differentiation (65). As with cDCs, mo-DCs have been identified in the TME of mice (66) and human tumor ascites (65), and may also contribute to anti-tumor immunity as they were suggested to actively suppress T-cell responses (66). Indeed, anthracycline chemotherapy can prompt massive recruitment and differentiation of monocytes. In this model the therapeutic benefit of chemotherapy relied on CD11b^+^ cells (67), suggesting that these mo-DCs may exhibit anti-tumor activity. Many questions remain as to how mo-DCs develop, if mo-DC populations from these studies share common transcriptional programs, and how they are functionally distinct from their peer cDCs. While seemingly semantic, clarity on origin, and functional specification will allow for more consistent comparisons across models and shed light on the myeloid populations that contribute to anti-tumor responses.

### Plasmacytoid DCs

Plasmacytoid DCs (pDCs) develop from the common DC progenitor (CDP), but are independent of the cDC lineage (47). While they can promote antiviral responses through type-I interferon, pDCs can also induce tolerance and have been associated with poor outcome in breast and ovarian cancer (68). Despite their proposed tolerogenic properties, however, some studies have found potent anti-tumor activity in pDCs upon therapeutic stimulation (68). It is important to note, however, that a recent study identified human CD123^+^ CD33^+^ pre-cDCs to exhibit substantial surface marker overlap with pDCs (69). Although CD33 and several other markers can separate pre-cDCs from pDCs (69), the cells used in older studies of pDCs may be contaminated with pre-cDCs, and conclusions drawn may warrant reevaluation.

### Monocytes

In both mouse and human, monocytes develop in a colony-stimulating factor 1 (CSF-1) dependent manner primarily in the bone marrow, through differentiation of monocyte-committed common monocyte progenitor (cMoP) population (70, 71). Although single-cell sequencing approaches are rapidly identifying subsets of these cells in bone-marrow, blood, and tissue, two primary subtypes, classical and non-classical or “patrolling” monocytes, clearly exist within the blood (72, 73).

In human, classical monocytes are characterized by their expression of both CD14 and CD16, while in mouse they are described as being Ly6C^hi^CCR2^+^. Classical monocytes, hereby referred to as Ly6C^hi^ monocytes, are thought to persist in circulation for 1–2 days, at which point they have either entered a tissue site in response to a stimulus, differentiated into a non-classical monocyte, or died (74). Studies on a population of cells known as monocytic myeloid-derived suppressor cells (mMDSC) (75), which includes monocytes, have been shown to promote tumor growth through the production of various immunosuppressive cytokines and factors (76–78) and the suppression T-cell proliferation and function (79), suggesting that perhaps, even as an undifferentiated precursor monocytes may contain functional capacity of consequence. Furthermore, a recent study using multiphoton intravital imaging of the lung pre-metastatic site in mice revealed that as pioneering metastatic tumor cells arrived and died, distinct waves of myeloid cells ingested tumor material, supplying antigen to both pro- and anti-tumor immune compartments (80). Monocytes were found to engulf the majority of tumor material, potentially sequestering valuable tumor antigen from stimulatory DC populations and genetic ablation of monocytes resulted in higher antigen loads in those DC.

Non-classical monocytes, hereby referred to as Ly6C^low^ monocytes, are described in human as being CD14^low^ CD16^+^ and in mouse as Ly6C^low^CCR2^−^Nr4a1^+^ (81). Unlike their Ly6C^hi^ monocyte counterparts, the function and critical features of Ly6C^low^ monocytes are poorly understood, particularly in the TME. Ly6C^low^ monocytes are generally characterized as being blood-resident, which helps explain data suggesting a role for them in the surveying of endothelial integrity (82, 83). While the role and even presence of Ly6C^low^ monocytes in the TME is debatable, Ly6C^low^ monocyte involvement in the metastatic site is clearer. A recent study (84) using Nr4a1-deficient mice, which lack Ly6C^low^ monocytes, demonstrated that in the absence of Ly6C^low^ monocytes tumor metastatic burden significantly increased but could be reduced by adoptive transfer of *Nr4a1*-proficient LyC6^low^ monocytes. It was shown that infiltrating Ly6C^low^ monocytes detect tumor through CX3CR1 and were capable of phagocytosing tumor material.

### Granulocytes

These cells include tumor associated **neutrophils** (TANs), which are distinct from circulating neutrophils in phenotype, cell surface markers, and chemokine activity. These neutrophils are recruited to the tumor site through various chemokine and receptor systems and their accumulation in the tumor is influenced by multiple factors and interactions with other cells types and environmental cues in the TME (85). Although neutrophils can inhibit or promote tumor progression based on their active role as regulators of the immune system and their impact on the TME, clinical evidence show their correlation with poor prognosis in multiple tumor indications such as melanoma, lung, melanoma, and renal carcinomas (86–90). Various reviews have highlighted the paradoxical role of neutrophils and provided insights on the mechanisms for their recruitment to the tumor site, their functional plasticity and polarization, and their activation to support tumor progression or enhance their antitumor functions (91–97). At present, numerous laboratories are engaged in single-cell sequencing efforts focusing on neutrophil heterogeneity, polarization, and lineage determination.

Beyond neutrophils, inflamed tissue can contain **mast cells, eosinophils, or basophils**, but little is known of the possible role(s) for these cells in cancer progression and the surrounding microenvironment. Similar to monocytes and macrophages, these cells can produce various angiogenic and lymphangiogenic factors important for tumor development and metastasis, chronic inflammation, and tissue injury and remodeling (98, 99). Mast cells are the stromal components of the inflammatory microenvironment and secrete a myriad of protumorigenic and antitumorigenic molecules depending on the environment, the tumor type, or their peritumoral or intratumoral localization (100). Eosinophils and basophils can also have protumorigenic or antitumorigenic roles, depending on their modulatory and regulatory functions to other immune cells in the TME or to their cytotoxic effects against tumor cells (101–103). Increasing evidence suggests that neutrophils, mast cells, eosinophils, or basophils can be potential therapeutic targets in different types of tumors (3, 100). However, there are still many unanswered questions that should be addressed before we understand their exact function in tumor progression and design accurate strategies for targeting them.

Both monocytes and neutrophils are often referred to as “myeloid-derived suppressor cells” or MDSC, a name given based on data suggesting a pro-tumoral, immune suppressive function when cultured with T-cells. For clarity purposes, we will refer to all myeloid cells in this review based on their individual population name.

## Therapeutic Targeting of Myeloid Populations in the TME

**Figure 2:**
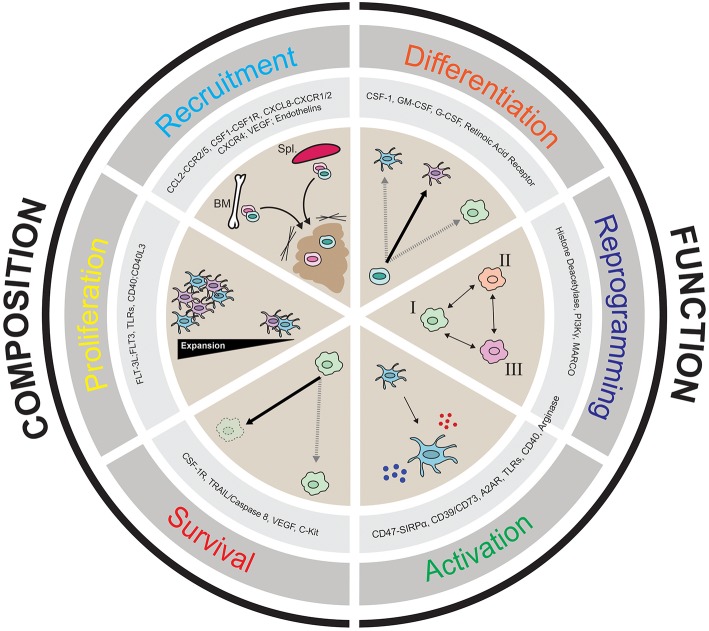
Cartoon depicting the six **“Pillars of Myeloid Tuning”** and the myeloid targets within each category. The myeloid tuning strategies affecting the **Composition** of the TME include targets modulating Recruitment (CCL2-CCR2/5, CSF1-CSF1R, CXCL8-CXCR1/2; CXCL12-CXCR4, VEGF-VEGFR, Endothelins), Proliferation (FLT3L-FLT3, TLRs, CD40-CD40L), and Survival (CSF1R, TRAIL/Caspase 8, VEGF, c-kit). The myeloid tuning strategies altering the **Function** of the TME include targets inducing Differentiation (CSF1, GM-CSF, G-CSF, Retinoic Acid Receptor ATRA), Reprogramming (Histone Deacetylase, CSF1R, MARCO, Arginase, PI3Kγ), and Activation (CD47-SIRPα, A2AR, CD73/CD39, STING, TLRs, CD40, Arginase). The indicated myeloid targets in each category are not comprehensive.

Given that there are populations of myeloid cells within the TME that impede productive anti-tumor immunity, it is of great interest to target myeloid populations that block anti-tumor immunity antagonistically, or to activate stimulatory cells that can help promote anti-tumor immunity. In this review we discuss the notion of “myeloid tuning,” which broadly involves the use of therapeutic compounds to change the **composition of myeloid cells** in the TME or to alter **their functional attributes**. Figure 2 describes the six myeloid tuning strategies and highlights the myeloid targets known to inhibit recruitment, block survival, affect proliferation, induce immune activation, alter differentiation, and stimulate reprogramming of myeloid cells in the TME (Figure 2 and Table 1). Multiple recent reviews have described various strategies to target the myeloid cells in the TME (3, 25, 29, 30, 38, 104–106). Here we aim to focus on the ongoing clinical trials of agents targeting the TME myeloid cell populations that are showing early therapeutic promise, placing them within the “myeloid tuning” mechanisms-of-action framework.

**Table 1:** Summary of ongoing clinical trials with agents that target myeloid cells.

**Target**	**Myeloid target cells**	**Drug name**	**Drug class**	**Sponsor**	**Indications**	**Clinical trials**
CCL2	Chemokine for Monocytes and other immune cells	NOX-E36	PEG-Aptamer	Noxxon Pharma AG	NSCLC and PDAC	Phase Ib/II Planned
CCR2 CCR5	Monocytes Macrophages, DCs, T-cells	BMS-813160	SM	Bristol-Myers Squibb Co	Advanced Solid Tumors	Phase Ib/II, NCT03184870
CCR2	Monocytes Macrophages, DCs, T-cells	CCX872-B	SM	ChemoCentryx Inc	Metastatic Adenocarcinoma of the Pancreas	Phase Ib; NCT02345408
CXCR2	Neutrophils, Mast cells, Macrophages, Monocytes	SX-682	SM	Synthrix Biosystems Inc	Metastatic Melanoma	Phase I; NCT03161431
CXCL8	Chemokine for neutrophils and monocytes	BMS-986253	SM	Bristol-Myers Squibb Co	Hormone sensitive prostate cancer	Phase Ib/II; NCT03689699
CSF1R	Monocytes, Macrophages, DCs (also on microglia, Paneth cells, Ostecolasts, Epithelial cells)	PLX-3397	SM	Plexxikonn Inc	Tenosynovial Giant Cell Tumor	Phase III NCT02371369
CSF1R	Monocytes, Macrophages, DCs (also on microglia, Paneth cells, Ostecolasts, Epithelial cells)	PLX-3397	SM	Plexxikonn Inc	Solid Tumors GBM and Gliosarcoma Refractory Leukemias and Refractory Solid Tumors KIT-mutated Melanoma Metastatic Breast Cancer	Phase I; NCT01004861 Phase I/II; NCT02777710 Phase I/II; NCT01790503 Phase I/II; NCT02390752 Phase I/II; NCT02975700 Phase Ib/II; NCT01596751
CSF1R	Monocytes, Macrophages, DCs (also on microglia, Paneth cells, Ostecolasts, Epithelial cells)	ARRY-382	SM	Array BioPharma Inc	Solid Tumors	Phase Ib/II NCT02880371
CSF1R	Monocytes, Macrophages, DCs (also on microglia, Paneth cells, Ostecolasts, Epithelial cells)	Cabiralizumab	mAb	Bristol-Myers Squibb Co	Advanced Pancreatic Cancer Stage IV Pancreatic Cancer Resectable Biliary Tract Cancer	Phase II; NCT03336216 Phase II; NCT03697564 Phase II; NCT03768531
CSF1R	Monocytes, Macrophages, DCs (also on microglia, Paneth cells, Ostecolasts, Epithelial cells)	Cabiralizumab	mAb	Bristol-Myers Squibb Co	Advanced Melanoma, NSCLC, and RCC Tenosynovial Giant Cell Tumor Selected advanced cancers	Phase I; NCT03502330 Phase I/II; NCT02471716 Phase I; NCT02526017
CSF1R	Monocytes, Macrophages, DCs (also on microglia, Paneth cells, Ostecolasts, Epithelial cells)	BLZ-945	SM	Novartis AG	Advanced Solid Tumors	Phase I/II; NCT02829723
CSF1R	Monocytes, Macrophages, DCs (also on microglia, Paneth cells, Ostecolasts, Epithelial cells)	LY-3022855	mAb	Eli Lilly and Co	Melanoma Pancreatic Cancer	Phase I/II; NCT03101254 Phase I; NCT03153410
CSF1R	Monocytes, Macrophages, DCs (also on microglia, Paneth cells, Ostecolasts, epithelial cells)	Emactuzumab	mAb	F. Hoffmann-La Roche Ltd	Advanced Solid Tumors Platinum-Resistant Ovarian Cancer	Phase I; NCT02323191 Phase II; NCT02923739
CSF1R	Monocytes, Macrophages, DCs (also on microglia, Paneth cells, Ostecolasts, Epithelial cells)	AMG-820	mAb	Amgen	Pancreatic Cancer, Colorectal Cancer, NSCLC	Phase Ib/II; NCT02713529
CSF1R	Monocytes, Macrophages, DCs (also on microglia, Paneth cells, Ostecolasts, Epithelial cells)	DCC-3014	SM	Deciphera Pharmaceuticals LLC	Hematological Tumors; Solid Tumors	Phase I; NCT03069469
CSF1R	Monocytes, Macrophages, DCs (also on microglia, Paneth cells, Ostecolasts, Epithelial cells)	SNDX-6352	SM	Syndax Pharmaceuticals Inc	Solid Tumors	Phase I; NCT03238027
M-CSF	Growth factor for monocytes, macrophages, and other cells	Lacnotuzumab	mAb	Novartis AG	Advanced Malignancies	Phase Ib/II; NCT02807844
M-CSF	Growth factor for monocytes, macrophages, and other cells	PD-0360324	mAb	Pfizer Inc	Platinum-Resistant Epithelial Ovarian Cancer	Phase II; NCT02948101
CD47	Tumor Cells, Red Blood Cells	Hu-5F9G4	mAb	Forty-Seven Inc	Hematological Malignancies Relapsed/Refractory B-cell Non-Hodgkin's Lymphoma Ovarian Cancer Solid Tumors and Advanced Colorectal Cancer	Phase I; NCT03248479 Phase Ib/II; NCT02953509 Phase I; NCT03558139 Phase Ib/II; NCT02953782
CD47	Tumor Cells, Red Blood Cells	IBI-188	mAb	Innovent Biologics Inc	Advanced Malignant Tumors and Lymphoma Advanced Malignancies	Phase I: NCT03763149 Phase I: NCT03717103
CD47	Tumor Cells, Red Blood Cells	CC-90002	mAb	Celgene Corp	Advanced Solid and Hematologic Cancers	Phase I; NCT02367196
CD47	Tumor Cells, Red Blood Cells	SRF-231	mAb	Surface Oncology Inc	Advanced Solid and Hematologic Cancers	Phase I; NCT03512340
SIRPα	Macrophages, DCs	ALX-148	Fusion protein	ALX Oncology Inc	Advanced Solid Tumors and Lymphoma	Phase I; NCT03013218
SIRPα	Macrophages, DCs	TTI-621	Fusion protein	Trillium Therapeutics Inc	Hematologic Malignancies and Selected Solid Tumors Relapsed and Refractory Solid Tumors	Phase I; NCT02663518 Phase I; NCT02890368
SIRPα	Macrophages, DCs	TTI-622	Fusion protein	Trillium Therapeutics Inc	Relapsed or Refractory Lymphoma or Myeloma	Phase I; NCT03530683
PI3Kγ	Macrophages, neutrophils, eosinophils. Mast cells	IPI-549	SM	Infinity Pharmaceuticals Inc	Advanced Solid Tumors Advanced HPV+ and HPV- HNSCC	Phase I; NCT02637531 Phase II; NCT03795610
A2AR	T-cells, monocytes, macrophages, DCs, NKs	CPI-444	SM	Corvus Pharma	Advanced Cancers	Phase I; NCT02655822
A2AR	T-cells, monocytes, macrophages, DCs, NKs	PBF-509	SM	Novartis AG	Advanced NSCLC	Phase I; NCT02403193
A2AR	T-cells, monocytes, macrophages, DCs, NKs	AB-928	SM	Arcus Biosciences Inc	Advanced Malignancies Gastrointestinal Malignancies TNBC and Gynecologic Malignancies Lung Cancer	Phase I; NCT03629756 Phase I; NCT03720678 Phase I; NCT03719326 Phase I; NCT03846310
CD73	Ectonucleotidase in the TME	MEDI-9447	mAb	MedImmune LLC	Advanced EGFRm NSCLC Relapsed Ovarian Cancer Metastatic Triple-Negative Breast Cancer Metastatic Pancreatic Cancer	Phase Ib/II; NCT03381274 Phase II; NCT03267589 Phase I/II; NCT03616886 Phase Ib/II; NCT03611556
CD73	Ectonucleotidase in the TME	CPI-006	mAb	Corvus Pharma	Advanced Cancers	Phase I; NCT03454451
CD73	Ectonucleotidase in the TME	BMS-986179	mAb	Bristol-Myers Squibb Co	Advanced Solid Tumors	Phase I/IIa; NCT02754141
CD73	Ectonucleotidase in the TME	AB-680	SM	Arcus Biosciences Inc	Healthy Volunteers	Phase I; NCT03677973
CD73	Ectonucleotidase in the TME	NZV-930	mAb	Novartis AG	Advanced Malignancies	Phase I; NCT03549000
Arginase	Macrophages, Neutrophils	CB-1158	SM	Calithera/Incyte Corp	Advanced and Metastatic Solid Tumors Relapsed or Refractory Multiple Myeloma	Phase I/II; NCT02903914, NCT03314935 Phase I/II; NCT003837509
Arginase	Macrophages, Neutrophils	AEB-1102	Rec Enzyme	Aeglea Biotherapeutics	Advanced Solid Tumors Extensive Disease SCLC	Phase I; NCT02561234 Phase I/II; NCT03371979
Arginase	Macrophages, Neutrophils	ARG1-18	Vaccine	Herlev Hospital	Metastatic Solid Tumors	Phase I; NCT03689192
TLR3	DCs, Macrophages, T-cells	Rintatolimod	Oligonucleotide	Hemispherx Biopharma Inc	Recurrent Ovarian Cancer Metastatic Colorectal Cancer Peritoneal Surface Malignancies Metastatic TNBC	Phase II; NCT03734692 Phase I; NCT03403634 Phase I/II; NCT02151448 Phase I; NCT03599453
TLR4	Macrophages, Monocytes, Granulocytes, DCs	G100	Rec Adenovirus	Immune Design Corp	Follicular Non-Hodgkin's Lymphoma Cutaneous T-cell Lymphoma	Phase I/II; NCT02501473 Phase II; NCT03742804
TLR4	Macrophages, Monocytes, Granulocytes, DCs	GSK-091	SM	GlaxoSmithKline Plc	Advanced Solid Tumors	Phase I; NCT03447314
TLR4	Macrophages, Monocytes, Granulocytes, DCs	ECI-006	Oligonucleotide	eTheRNA Immunotherapies	Metastatic Melanoma	Phase I; NCT03394937
TLR5	Macrophages, Monocytes, DCs, T-cells, Intestinal Epithelial cells	M-VM3	Vaccine	Panacela Labs Inc	Prostate Cancer	Phase Ib; NCT02844699
TLR7	B-cells, DCs, Monocytes, Macrophages, Neutrophils	Imiquimod UGN-102	SM	UroGen Pharmaceuticals Ltd	Non-muscle Invasive Bladder Cancer (NMIBC)	Phase II; NCT03558503
TLR7TLR8	B-cells, DCs, Monocytes, Macrophages, Neutrophils	NKTR-262	SM	Nektar Therapeutics	Advanced or Metastatic Solid Tumor Malignancies	Phase I/II; NCT03435640
TLR7 TLR8	B-cells, DCs, Monocytes, Macrophages, Neutrophils	Resiquimod R848	SM	Galderma SA	Metastatic Melanoma	Phase II; NCT00960752
TLR8	DCs, Monocytes, Macrophages, Neutrophils	Motolimod VTX-2337	SM	Celgene Corp	Recurrent, Platinum-Resistant Ovarian Cancer	Phase I/II; NCT02431559
TLR9	B-cells, T-cells, Macrophages, Monocytes, Neutrophils	Lefitolimod MGN1703	Oligonucleotide	Mologen AG	Metastatic Colorectal Cancer Advanced Solid Tumors	Phase III; NCT02077868 Phase I; NCT02668770
TLR9	B-cells, T-cells, Macrophages, Monocytes, Neutrophils	Tilsotolimod	Oligonucleotide	Idera Pharmaceuticals Inc	Solid Tumors	Phase II; NCT03865082
TLR9	B-cells, T-cells, Macrophages, Monocytes, Neutrophils	AST-008	Oligonucleotide	Exicure Inc	Advanced Solid Tumors	Phase Ib/II; NCT03684785
TLR9	B-cells, T-cells, Macrophages, Monocytes, Neutrophils	CMP-001	Oligonucleotide	Checkmate Pharmaceuticals Inc	Metastatic Colorectal Cancer Non-small Cell Lung Cancer Advanced Melanoma Melanoma with LN disease	Phase I; NCT03507699 Phase I; NCT03438318 Phase I; NCT02680184 Phase II; NCT03618641
TLR9	B-cells, T-cells, Macrophages, Monocytes, Neutrophils	SD-101	Oligonucleotide	Dynavax Technologies Corp	Relapsed or Refractory Follicular Lymphoma B-Cell Non-Hodgkin Lymphoma Advanced or Metastatic Solid Malignancies	Phase Ib/II; NCT02927964 Phase I; NCT03410901 Phase I; NCT03831295
TLR9	B-cells, T-cells, Macrophages, Monocytes, Neutrophils	DV-281	Oligonucleotide	Dynavax Technologies Corp	Non-small Cell Lung Carcinoma	Phase I; NCT03326752
DC	DCs	Poly-ICLC (Hiltonol)	Vaccine	Oncovir Inc	MRP Colon Cancer Unresectable Solid Cancers Recurrent Pediatric Gliomas Solid Cancer Prostate Cancer	Phase I/II; NCT02834052 Phase I/II; NCT03721679 Phase II; NCT01188096 Phase II; NCT02423863 Phase I; NCT0362103
FLT3L	DC Progenitors, pDCs, cDCs	rhuFlt3L/(CDX-301)	Rec protein	Celldex Therapeutics	Low Grade B-Cell Lymphomas Advanced NSCLC	Phase I/I; NCT01976585 Phase II; NCT02839265
STING	T-cells, NK cells, DCs, Monocytes, Macrophages	MK-1454	SM	Merck & Co Inc	Advanced/Metastatic Solid Tumors and Lymphomas	Phase I; NCT03010176
STING	T-cells, NK cells, DCs, Monocytes, Macrophages	ADU-S100 (MIW815)	SM	Aduro BioTech Inc	Advanced/Metastatic Solid Tumors and Lymphomas	Phase I; NCT02675439 NCT03172936
CD40	DCs, Macrophages, Monocytes, B-cells, Endothelial Cells, Tumor Cells	APX-005M	mAb	Apexigen Inc	Solid Tumors Advanced Sarcomas Metastatic Melanoma Pediatric CNS Tumors Metastatic Pancreatic Cancer	Phase I; NCT02482168 Phase II; NCT03719430 Phase I/II; NCT02706353 Phase I; NCT03389802 Phase Ib/II; NCT03214250
CD40	DCs, Macrophages, Monocytes, B-cells, Endothelial Cells, Tumor Cells	Selicrelumab	mAb	F. Hoffmann-La Roche Ltd	Advanced/Metastatic Solid Tumors	Phase I; NCT02665416, NCT02304393
CD40	DCs, Macrophages, Monocytes, B-cells, Endothelial Cells, Tumor Cells	ABBV-927	mAb	AbbVie Inc	Advanced Solid Tumors Advanced Head and Neck Cancer	Phase I; NCT02988960 Phase I; NCT03818542
CD40	DCs, Macrophages, Monocytes, B-cells, Endothelial Cells, Tumor Cells	MEDI-5083	Fusion protein	MedImmune LLC	Advanced Solid Tumors	Phase I; NCT03089645
CD40	DCs, Macrophages, Monocytes, B-cells, Endothelial Cells, Tumor Cells	SEA-CD40	mAb	Seattle Genetics Inc	Advanced Malignancies	Phase I; NCT02376699
CD40	DCs, Macrophages, Monocytes, B-cells, Endothelial Cells, Tumor Cells	JNJ-7107 (ADC-1013)	mAb	Johnson & Johnson	Advanced Stage Solid Tumors	Phase I; NCT02829099
CD40	DCs, Macrophages, Monocytes, B-cells, Endothelial Cells, Tumor Cells	CDX-1140	mAb	Celldex Therapeutics Inc	Advanced Malignancies	Phase I; NCT03329950

### Targets and Therapies That Alter TME Myeloid Population Composition by Altering Cell Recruitment, Proliferation, and Survival

Altering the recruitment of specific subsets of myeloid cells to the tumor, or modulating their proliferation or survival is viewed as a promising approach to promote durable anti-tumor responses either as single agent therapies or in combination with currently available cancer therapies. Many of the myeloid protein targets that are being pursued therapeutically to alter TME myeloid composition (Figure 2) vary in their specificity or lack thereof for specific subsets, and are discussed below.

#### CCL2-CCR2 Axis

The chemokine CCL2 and its receptor CCR2 are critical for attracting monocytes into tissues. CCR2 inhibition retains monocytes in the bone marrow and reduces the number of TAMs in tumors, leading to decreased tumor burden and metastasis in different tumor indications (107–114). Previous reviews have described different strategies to prevent CCL2-mediated recruitment of myeloid cells and elucidated the pharmacological difficulties in safely and efficiently blocking this CCL2/CCR2 axis (29, 30, 115–118). Multiple experimental agents targeting the CCL2/CCR2 axis also showed limited efficacy in the clinic, and the clinical testing of some of these agents were recently discontinued (e.g., Carlumab from Centocor/J&J, Plozalizumab from Millenium Pharamceuticals, and PF-04136309 from Pfizer). The limited therapeutic efficacy of the Carlumab was attributed to the profound accumulation of total CCL2 in the periphery due to high chemokine synthesis rate and the significant discrepancy between the *in vitro* and *in vivo* K_D_ values (119, 120). The limited efficacy and lack of durable responses of these agents could in part be linked to the rapid compensation by granulocytes, the lack of effect on tissue resident macrophages, and the rebound in monocyte recruitment after treatment cessation as seen in pre-clinical models (37, 121, 122). The anti-CCR2 mAb Plozalizumab was terminated in a Phase I trial in malignant melanoma (NCT02723006) due to a classified business decision in May 2018. PF-04136309, a small molecule antagonizing CCR2, was used in combination with FOLFIRINOX in a Phase Ib study in resectable pancreatic ductal carcinoma (NCT01413022). Treatment related toxicities of grade ≥3 adverse events were seen in ≥10% of patients treated with both therapies, which included neutropenia, lymphopenia, hypokalemia, and diarrhea (123). Another clinical trial in metastatic pancreatic patients using PF-04136309 in combination with nab-paclitaxel and gemcitabine was terminated in May 2017 (NCT02732938) reported by the sponsor as due to a change in portfolio strategy without commenting on either safety or efficacy signals. Previously, it had been reported that in 21 enrolled patients, the drug had encouraging safety, PK, and efficacy profiles (124).

NOX-E36, an Emapticap pegol RNA Aptamer that targets CCL2 showed an acceptable clinical safety profile in type II diabetes patients and decreased the CCR2+ monocytes blood count as expected [NCT01547897; (125)]. NOX-E36 therapy in a mouse tumor model inhibited the infiltration of tumor-associated macrophages leading to significant changes of the TME and a reduction in liver tumor burden (126). The small molecule inhibitor CCX-872, which targets CCR2, is currently in the clinic for the treatment of patients with advanced and metastatic pancreatic cancer (NCT02345408), and data from the ongoing Phase Ib trial demonstrated promising safety and overall survival with the CCX872 and FOLFIRINOX combination therapy compared to FOLFIRINOX alone (127, 128).

#### CCL5-CCR5 Axis

Notably, alternative recruitment of monocytes can be achieved via the CCL5-CCR5 axis (129) and inhibiting that axis also restricted cancer growth in colorectal cancer (130) and blocked metastasis of basal breast cancer cells. A dual small molecule inhibitor, BMS-813160, that targets both CCR2 and CCR5, is being tested in patients with advanced pancreatic cancer in combination with Nivolumab and Gemcitabine and Nab-paclitaxel (NCT03184870).

Emerging data suggest that tumor-produced **IL-8** (CXCL8) plays an important role in recruiting neutrophils and monocytes into the TME of many cancer types (131). Neutralization of IL-8 by the mAb HuMax-IL8 in TNBC decreases the recruitment of neutrophils (also referred to as PMN-MDSCs) to the tumor site and facilitates immune-mediated killing (132). The IL-8 inhibitor BMS-986253 is being tested in a Phase Ib/II trial in combination with Nivolumab in hormone-sensitive prostate cancer [NCT03689699; (133)].

#### CSF1-CSF1R Axis

The CSF1/CSF1R axis plays a key role in the differentiation, recruitment, proliferation, and survival of both monocytes and macrophages (134). Multiple inhibitors of the CSF1/CSF1R axis are being clinically developed, and these inhibitors have been extensively reviewed (29, 135–137). The most advanced agent in clinical testing is the small molecule selective kinase inhibitor Pexidartinib (PLX-3397), which is being tested in a Phase III trial in Tenosynovial Giant Cell Tumors (TGCT; NCT02371369). Pexidartinib demonstrated efficacy in TGCT (136, 138, 139). TGCT is driven by the translocation of chromosome 1 and 2 (1p13 to 2q35), which leads to the overexpression of CSF1 caused by the fusion of *CSF1* to *COL6A3* (140). Pexidartinib is also being investigated for the treatment of various solid tumors, such as metastatic breast cancer, advanced ovarian cancer, colorectal cancer, and pancreatic cancer, in combination with chemotherapy or ICBs (Table 1). In pre-clinical models, PLX-3397 increased the efficacy of anti-PD-1 or chemotherapy treatments (141, 142). While PLX-3397 is a CSF1R inhibitor, it also targets the c-kit and FLT3 receptor tyrosine kinases (RTKs), which are expressed on other myeloid populations including mast cells and dendritic cells. Two other small molecules CSF1R inhibitors are in development, BLZ-945 and ARRY-382. BLZ-945 is currently in Phase I/II trails for patients with advanced solid tumors (NCT02829723). In preclinical studies, BLZ-945 was shown to repolarize TAMs to become antitumoral in mouse models of glioblastoma by downregulating genes that have been associated with an M2-like macrophage polarization phenotype (143), and to decrease tumor progression as monotherapy and in combination with ICBs in a mouse model of neuroblastoma (144). ARRY-382 is also being tested in Phase I/II in patients with advanced solid tumors (NCT02880371). Preliminary clinical data demonstrated partial responses with a manageable tolerability profile (145).

The anti-CSF1R mAb, Cabiralizumab blocks the activation and survival of monocytes and macrophages by inhibiting the binding of the two ligands CSF1 and IL-34 to CSF1R (146, 147). Cabiralizumab is being tested in a Phase I clinical trial in advanced solid tumors (NCT02526017), in a Phase II trial in advanced pancreatic cancer (NCT03336216), and in TGCT (NCT02471716). Preliminary data suggests tolerable safety profiles in combination with Nivolumab and durable clinical benefits in heavily pretreated patients with pancreatic cancer (148). Recent data showed that treatment with Cabiralizumab and Nivolumab depletes immunosuppressive TAMs and promotes a pro-inflammatory TME (149). For instance, tumors from treated patients had a decrease in CSF1R+ macrophages, an increase in CD8+ T cells, and an increase in pro-inflammatory genes. Moreover, these patients had increased levels of CSF1/IL-34 and decreased levels non-classical monocytes in the periphery (149). In addition, Cabiralizumab demonstrated initial clinical benefits in patients with Pigmented Villonodular Synovitis (150). In addition to Cabiralizumab, several other antagonistic anti-CSF1R mAbs are in clinical development (see Table 1). AMG-820 (a fully human IgG2 targeting CSF1R) resulted in a 32% stable disease in a Phase II study (NCT01444404) in patients with relapsed or refractory advanced solid tumors and induced adverse effects including periorbital edema, increased aspartate aminotransferase, fatigue, nausea, blurred vision, and deafness (151). AMG-820 is also being tested in combination with pembrolizumab in patients with pancreatic, NSCLC, and colorectal cancer (NCT02713529). LY3022855 (a humanized IgG1 targeting CSF1R) is being tested in a Phase I/II trial in patients with metastatic melanoma in combination with BRAF/MEK inhibitors (NCT03101254). Emactuzumab is a mAb that blocks CSF1R dimerization, and demonstrated a 7% complete response rate in a Phase I trial with TCGT patients and had no reported dose toxicity (152). Targeting the CSF1R ligand CSF1 has also proven to be a promising strategy. Two mAbs developed by Novartis (Lacnotuzumab) and Pfizer (PD-0360324) are currently in the clinic. Recent data from Lacnotuzumab (MCS110)'s Phase Ib clinical trial in advanced malignancies showed it is tolerated and has preliminary antitumor activity, especially in the pancreatic cancer cohort. However, grade 3 suspected drug-related adverse effects were observed and included periorbital edema, increased blood creatine phosphokinase, and increased aspartate aminotransferase (AST) (153).

While targeting the CSF1/CSF1R axis has shown clinical promise, novel resistance, and compensatory mechanisms could emerge. For instance, acquired and inherent resistance to CSF1R blockade has been reported in pre-clinical mouse models of glioblastoma multiforme and other cancer types harboring specific genetic alterations (105). Moreover, a recent study identified a compensation between CSF1R+ macrophages and Foxp3+ regulatory T-cells (Tregs) that can drive resistance to immunotherapy in a mouse model of colorectal cancer (154). In addition, the common side effects observed in most of the CSF1/CSF1R antagonistic small molecules and mAbs developed could be caused by the systemic depletion of tissue resident macrophages in normal tissues. In addition to targeting the CSF1/CSF1R axis to reduce tumor associated myeloid cells there are a number of additional agents, including trabectidin (Yondelis®), lurbinectedin, and the bisphosphonates clodronate and zoledronic acid (3, 29, 116). While there are multiple ongoing clinical trials to evaluate bisphosphonates, there is no available data regarding their anti-tumor activity. Therefore, finding new targets that are selectively upregulated in the TAMs and tumor-associated monocytes is crucial and might lead to more clinical benefits with fewer side effects.

### Targets and Therapies That Alter TME Myeloid Population Function by Altering Cell Activation, Reprograming, and Differentiation

Altering the activation status of pro-tumorigenic myeloid cells to inhibit their immunosuppressive activity (reversal of immunosuppression) or altering pro-tumorigenic myeloid cells by differentiating or reprograming them to become anti-tumorigenic is viewed as another promising approach to promote durable anti-tumor responses either as single agent therapies or in combination with currently available cancer therapies. Another approach to alter the TME myeloid population function is to induce activation of anti-tumorigenic myeloid cells such as DCs. Many of the myeloid protein targets that are being therapeutically pursued to alter TME myeloid function are shown in Figure 2 and listed in Table 1 and are discussed below.

#### CD47-SIRPα Regulation of Phagocytosis

The CD47-SIRPα axis is a myeloid specific ICB that inhibits phagocytosis of tumor cells by macrophages and other myeloid cells (155). The “don't eat me signal” CD47 is overexpressed on the majority of hematopoietic malignancies and solid tumors and is also expressed on red blood cells (156–158). CD47 binds its ligand SIRPα, a RTK expressed on the cell surface of macrophages and dendritic cells and associates with the downstream inhibitory tyrosine phosphatases SHP-1 and SHP-2 (159). Recent review articles present the various clinical strategies to enhance phagocytosis by targeting the CD47-SIRPα axis (116, 155, 160, 161), and discuss the limitations and potential toxicities of targeting this axis (116, 160). The antagonistic anti-CD47 mAb Hu-5F9G4 induces phagocytosis of tumor cells by blocking the CD47-SIRPα interaction (162). Hu-5F9G4 was evaluated in a Phase Ib dose escalation study in patients with relapsed/refractory non-Hodgkin lymphoma, follicular lymphoma and diffuse large B-cell lymphoma (DLBCL) in combination with rituximab (NCT02953509). In this small study of 22 subjects (with 21/22 known to be refractory to single agent rituximab), anti-tumor responses were observed in 50% of subjects (including 36% with complete response). Hu-5F9G4 in combination with rituximab at standard rituximab doses was generally safe and a maximum tolerated dose of the antibody was not declared (163). The main on- target side effect was anemia, which could be mitigated and managed by initially “priming” subjects with a 1 mg/kg dose of Hu-5F9G4 to eliminate aging red cells prior to introducing therapeutic intent dosing. Dose limiting toxicities not requiring treatment discontinuation were reported in two subjects (pulmonary embolism and grade 4 neutropenia requiring G-CSF) (163). A third subject developed idiopathic thrombocytopenic purpura treated with glucocorticoids and immune globulin and required treatment discontinuation. Hu-5F9G4 is also being evaluated in patients with solid tumors (NCT02216409), and acute myeloid leukemia (AML) (NCT02678338) with and without azacitidine (NCT03248479). Recent data has shown this combination therapy leads to an increase in phagocytosis of AML blast cells by human macrophages *in vitro* and clearance of AML *in vivo*, leading to a prolonged survival compared to Hu-5F9G4 or azacitidine alone (164). Since anemia and neutropenia have been a concern for anti-CD47 therapies (165), strategies for better priming and maintenance doses are crucial. To this point, studies demonstrated that an initial priming dose of Hu-5F9G4 resulted in a near complete loss of CD47 antigen only on RBCs and not on white blood cells and AML bone marrow blasts, suggesting that CD47 pruning (loss) is protective for RBCs and could decrease the potential for toxicities (166). Hu-5F9G4 is also being evaluated in combination with Cetuximab in patients with solid tumors and advanced colorectal cancer in a Phase I/II study (NCT02953782). Clinical trials for another anti-CD47 mAb, CC90002, was recently terminated in patients with AML for unspecified hematologic toxicities described as reversible (NCT02641002), but it is still being tested in a Phase I dose escalation study in patients with other hematological cancers and advanced/refractory solid tumors followed by combination treatment with Rituximab (NCT02367196).

TTI-621, a SIRPα-Fc (human IgG1 Fc) fusion protein that blocks the CD47-SIRPα interaction, is being tested in patients with hematologic malignancies and solid tumors (NCT02663518), while TTI-622 (a SIRPα-Fc (human IgG4 Fc) fusion protein is being evaluated in a Phase I clinical trial in relapsed or refractory lymphoma or myeloma (NCT03530683). Currently it is unknown whether the SIRPα fusion proteins will have better efficacy and/or a better tolerability profile compared to the anti-CD47 mAb therapies.

#### Immunosuppressive Adenosine Signaling

Other strategies to activate the myeloid cells in the TME include the inhibition of their immunosuppressive functions, such as blocking the arginase, CD39/CD73 ectonucleotidases, and the adenosine A2A receptor (A2AR) pathways. Toward the latter, the extracellular adenosine concentrations and downstream signaling via the A2AR pathway has been shown to create a highly immunosuppressive microenvironment by significantly decreasing the immune responses in inflamed tissues and tumors (167–169). Many companies are developing mAbs and small molecules against these targets and some are being evaluated in the clinic (Table 1), and were recently reviewed (169). CPI-444 is a small molecule inhibitor targeting A2AR and is being evaluated in a Phase I trial in patients with advanced cancers (NCT02403193). Recent data from patients with refractory renal cell carcinoma showed that CPI-444 was well tolerated and prolonged survival as monotherapy and in combination with Atezolizumab (170). In addition, the expression of a novel adenosine biomarker signature in pre-treated tumor biopsies was significantly associated with tumor response to CPI-444 (171–173).

CD73 is the ectonucleotidase that catalyzes the irreversible conversion of AMP to adenosine, leading to the high levels of adenosine observed in the TME (174, 175). The monoclonal antibody MEDI-9447 (Oleclumab) antagonizes the enzymatic activity of CD73 through two distinct conformation-mediated mechanisms, which allows it to block both soluble and cell-surface CD73 in a non-competitive manner (176). MEDI-9477 can mediate changes in the infiltrating lymphoid and myeloid populations in the TME of mouse models, such as activation of macrophages and increasing CD8+ effector cells (177). In advanced pancreatic or colorectal cancer patients treated with Oleclumab (NCT0253774), free soluble CD73 and CD73 bound on peripheral T-cells were decreased across all doses and patients, and tumoral CD73 expression was also decreased (178). Oleclumab monotherapy and in combination with durvalumab showed manageable safety profile and encouraging clinical activity in colorectal and pancreatic cancer patients [NCT0253774; (178)]. While the adenosine pathway may be a key immunoregulatory node, we have to be prudently cautious about targeting specific members of the pathway without taking into account the biochemical pathway redundancies and feedback mechanisms that counter-regulate them.

#### TLRs and CD40 Agonists

Toll like receptors play important roles in the activation of the innate immune response and have been pivotal targets in cancer immunotherapy. They can selectively activate a subset of DC and macrophage populations to take on stimulatory and pro-inflammatory phenotypes (179–181). TLR3, TLR4, TLR5, TLR7, TLR8, and TLR9 agonists are being clinically evaluated (Table 1). The TLR7 agonist Imiquimod (topical cream) was approved for the treatment of basal cell carcinoma and showed additional efficacy in breast cancer skin metastases and melanoma. Imiquimod is believed to stimulate cytokine production, increase the infiltration of macrophages, DCs, and lymphocytes, and directly induce apoptosis in the tumor cells (182). Urogen Pharmaceuticals developed imiquimod (UGN-201) in a reverse thermal hydrogel formulated with the chemotherapeutic agent Mitomycin C (MMC), which is being evaluated in a Phase II trial in patients with low grade non-muscle invasive bladder cancer (NCT03558503). G100 is an intratumoral TLR4 agonist (composed of glucopranosyl lipid A in stable emulsion) that creates a systemic immune response when injected locally as a vaccine. G100 has been evaluated in multiple clinical trials and data from Phase I (NCT02501473) showed that it is well tolerated with clinical activity as a monotherapy and in combination with the anti-PD-1 antibody Pembrolizumab (183, 184). In addition, patients with TLR4 expression at baseline had a significant improved overall response rate (185). In a proof-of-concept trial in Merkel cell carcinoma patients (NCT02035657), intratumoral G100 induced anti-tumor immune responses leading to tumor regression without systemic toxicities (186). Based on encouraging results from a small early phase data, advanced trials are ongoing with intralesional SD-101 (a class c CpG Oligonucleotide TLR9 agonist) in combination with Pembrolizumab (187). The most advanced TLR9 agonist in the clinic is Lefitolimod (MGN1703), which is a synthetic DNA-based agonist that results in an antitumor immunomodulation, including increased release of cytokines and chemokines from peripheral blood mononuclear cells (PBMCs) and an increase in expression of surface activation markers of cells on a variety of immune cells (188–190). Lefitolimod is being evaluated in a pivotal Phase III trial of first-line maintenance in 549 enrolled patients with metastatic colorectal cancer (NCT02099868), following promising data in previous Phase I and II trials, where MGN1703 showed therapeutic efficacy in multiple solid tumors and was well-tolerated in long-term treatment with high doses (191–193).

The TNFR family member CD40 is expressed on the vast majority of myeloid cells such as DCs, macrophages, monocytes, and is also expressed on B-cells, tumor cells, and endothelial cells. Signals transduced by CD40 result in upregulation of multiple proteins critical to effector T-cell priming, including immunostimulatory cytokines, major histocompatibility (MHC) molecules, and the co-stimulatory ligands CD80 and CD86 (194–196). Multiple CD40 agonists have been developed to activate innate and adaptive immunity and some are being evaluated in the clinic [(197), Table 1]. APX-005M is the most advanced CD40 agonist in the clinic and is being tested in a Phase II trial in patients with advanced sarcomas (NCT03719430) and in Phase I/II in patients with metastatic melanoma [NCT02706353; (198)] and metastatic pancreatic cancer (NCT03214250). Recent preliminary data from the Phase Ib clinical trial in previously untreated metastatic pancreatic cancer showed that 20 out of the 24 patients had tumor shrinkage when treated with standard of care chemotherapy with and without Nivolumab. However, toxicity was a key concern as 13 out of the 24 patients experienced adverse effects and had to discontinue the treatment combinations (199). This trial has now progressed to the Phase II stage (NCT03214250).

#### Reprogramming Targets

Targets that are considered likely to induce switching in TAMs from a pro-tumoral to a tumoricidal state include class I and class II histone deacetylases (200–202), the macrophage receptor with collagenous structure MARCO (203), CD11b (204), and PI3Kγ (205, 206). Within these, PI3Kγ is a key regulator of the pro-tumoral and immunosuppressive state of TAMs and its genetic and pharmacological inhibition switches the TAMs to a pro-inflammatory state and subsequent tumor growth inhibition (205, 206). The selective small molecule PI3Kγ inhibitor IPI-549 was evaluated in a Phase I/Ib clinical trial in 220 patients with advanced solid tumors as monotherapy and in combination with Nivolumab (NCT02637531). IPI-549 was shown to be well-tolerated at all the doses tested and showed 40 percent disease control and durable partial responses in patients with indications not typically responsive to anti-PD1 therapy (207, 208). Data from peripheral blood from IPI-549 treated patients showed upregulation of IFN-gamma responsive factors and an increase in proliferation of exhausted memory T-cells (207, 208). In addition, paired tumor biopsies from monotherapy IPI-549 treated patients showed a decrease in CD163, sometimes called an “M2” macrophage marker (208), consistent with the mechanism-of-action in the pre-clinical studies of IPI-549 inducing immune activation and reducing immune suppression (205).

Many of the above discussed targets and drugs used in the clinic are not specific to specific subpopulations of myeloid cells and might be contributing to some of the side effects and toxicities discussed above. In order to identify novel targets specifically expressed on unique myeloid subsets, such as macrophages, neutrophils, and DCs, sophisticated technologies need to be employed. These include single-cell RNA-sequencing (scRNA-seq) and mass cytometry, and are discussed below.

## Identifying Novel TME Myeloid Subpopulations

To improve the efficacy and safety of agents that target myeloid subpopulations in the TME it will likely be necessary to have a deeper understanding of the extent of the functional diversity of intratumor myeloid subpopulations. Modern, high-throughput scRNA-seq, and cytometry by time of flight (CYTOF) technologies (209) have begun to revolutionize our understanding of the TME, both in terms of intra- and inter-tumoral variability. Historically, most efforts to understand the architecture and complexity of the TME were confined to the use of bulk RNA sequencing and microarray technologies which, while providing some sample and indication level differentiation, offer little insight into the cellular composition heterogeneity of an individual tumor. Granularity of gene expression associated with various stromal, malignant, and immune cell populations as well as any heterogeneity existing within those populations is lost upon averaging across cells to yield a single transcriptional profile. A variety of cellular deconvolution methodologies (210–214) were described to attempt to recapture this heterogeneity, but they rely on the existence of specific cellular markers that possess little or no collinearity between cell types. This approach works well for the major cellular constituents of a tissue but has limited efficacy in classifying subpopulations of cells or identifying rare, novel subsets. The capacity for new scRNA-seq methods to capture tens of thousands of unique, cellular transcriptomes in a single experimental run, particularly when combined with high-throughput flow cytometric sorting as an *a priori* enrichment strategy, offers a unique and powerful window into the TME. It enables not only the measurement of relative abundances of diverse cell types, but also the relationship, substructure and differentiation processes within those cells. Single cell methodologies now exist to profile mRNA, DNA, epitope levels, methylation, transcription factor binding, chromatin accessibility, and in some cases even preserving spatial information (215). Although insights and advances driven by single cell sequencing of the intratumoral myeloid compartment are, as of yet, limited, key lessons are beginning to emerge.

While a variety of human tumor ecosystems have been profiled at single cell resolution (21, 22, 216–223) only a few contain sufficient myeloid cells to adequately address questions of subpopulation heterogeneity, lineage dynamics, or ontogeny. To date, most studies interrogating the myeloid compartment of the TME focused specifically on macrophages, as they are, by far, the most abundant cell type in that milieu. In breast cancer, a positive correlation of M1- and M2-derived gene signatures across the aggregate of multiple subclusters of TAMs was shown (22) and identified a concomitant increase of M2-markers, MARCO, NRP2, and CD276 along with CCL3, sometimes associated with antitumoral functions, across macrophage lineages derived from trajectory-based analyses (22). These findings were corroborated in a study that performed single cell profiling of human gliomas, and correlated expression profiles of the M1-marker, TNFα, and the M2-marker, IL10, as evidence that a binary model of macrophage activation may not exist *in vivo* and instead may be better examined according to a spectrum-based model (222, 224). Similarly, the application of mass cytometry in clear cell renal cell carcinoma (220) revealed 17 separate TAM clusters, across which canonical *in vivo* differentiation markers exhibited a range of expression, not the expected binary distribution. Similar to activation status, macrophage ontogeny has been sparsely examined in the context of human single cell sequencing datasets. In IDH-mutant low-grade glioma (219) researchers found a spectrum of differentiation based on gene expression between tissue resident microglia and blood-derived macrophages whereas in late stage glioma, primarily glioblastoma, the two populations of macrophages appear quite distinct (222). Utilizing the aggregation of tumor and healthy cells to classify gene signatures or gene sets that differentiate TAMs from their tissue-resident brethren in a single lung adenocarcinoma patient, TREM2, MARCO, APOE, and CD81 were shown to be specifically upregulated in TAMs, relative to alveolar macrophages (21).

Investigation into the intratumoral complement of monocytes, dendritic cells, and granulocytes is, to this point, sorely lacking from a single cell sequencing perspective. This will undoubtedly improve, however, as cellular encapsulation technologies yield higher throughput and researchers begin to focus specifically on individual cellular populations via flow cytometric enrichment. This approach has already begun to yield dividends in the periphery, particularly in various dendritic cell populations with respect to ontogeny (46, 69) and the discovery of novel cellular subtypes (64). To this point, most single cell tumor studies have taken a macroscopic view of the tumor microenvironment: either all cells or partially enriched subsets are submitted for encapsulation and sequencing. Typically, samples from multiple patients are aggregated to generate sufficient numbers of cells to either differentiate between cell type or to provide a more global, indication-specific view of the tumor ecosystem. In this scenario, we urge researchers to also provide patient-specific analyses as the aggregation of samples homogenizes inter-patient variability in much the same way bulk sequencing homogenizes expression profiles across cell types. This issue is particularly important for human samples which, compared to tumors from in-bred mouse strains, are marked by extremely variable microenvironment composition.

Different issues arise when attempting to understand the heterogeneity within and between closely related cell types. Nearly all single cell technologies rely on the downstream identification of discrete cellular clusters. As recently reviewed by Andrew and Hemberg (225), these cluster identification algorithms range between K-means, hierarchical, graph, and density-based methods, each implemented in a variety of different ways. For divergent cell types that possess disparate functional programs, these methods generally converge. However, in the context of cells with a shared ontogeny, it can be quite difficult to arrive at a consistent pattern of clustering, particularly in light of the fact that most algorithms require *a priori* knowledge of resulting cluster number or require upfront modulation of parameters that directly dictate cluster number. In practice, this often means setting a fold-change cutoff that is reached by a set number of markers as the defining criterion for a cluster. The identification of a robust clustering of cells does not mean that those clusters have different biological and functional status. From an analytical standpoint, genes that differ between clusters may be assessed via gene set enrichment techniques to understand functional consequence and, of course, if those genes allow flow cytometric-based sorting, those populations may be compared with relevant experimental techniques.

## Conclusion

The different myeloid tuning strategies we discuss in this review describe the various myeloid targets and agents being investigated in the clinic. Some of these agents modulate the function of myeloid populations to inhibit their immunosuppressive activities and make them more anti-tumorigenic and some agents impact recruitment and survival of myeloid subpopulations. Few myeloid targeting strategies in the clinic have yielded promising results and many have been terminated due to toxicities related to the specificity or lack of tumor specificity of the target or to the properties of the agent being used. It is too early for us to know how these agents will play out in the clinic as many of the clinical trials are still ongoing and we have to wait for the results to determine their success or failure. However, the majority of the targets being pursued are not exclusively expressed on just one population of myeloid cells but rather they can be expressed on multiple myeloid populations, and even at times on lymphocytes and tumor cells.

While the understanding of intra- and intertumoral myeloid composition is in its nascent stages, particularly in humans, single cell sequencing technology will almost assuredly serve to identify heretofore unknown cellular subsets that may yield actionable targets in the fight against cancer. Additional pre-clinical studies are needed to determine the function of those novel targets in the TME and the pathophysiological relevance of the newly identified cellular cluster subsets. Finally, a more granular understanding of the kinetics and environmental queues that drive peripheral monocyte transition to TAM phenotypes could yield upstream targets designed to prevent the development of these type of suppressive cells.

## Author Contributions

All authors listed have made a substantial and intellectual contribution to the work, and approved it for publication.

### Conflict of Interest Statement

MK is a founder, board member, and shareholder at Pionyr Immunotherapeutics. NJ, JP, MB, VS, and LR are shareholders at Pionyr Immunotherapeutics. The remaining author declares that the research was conducted in the absence of any commercial or financial relationships that could be construed as a potential conflict of interest.
